# A New Powerful Nonparametric Rank Test for Ordered Alternative Problem

**DOI:** 10.1371/journal.pone.0112924

**Published:** 2014-11-18

**Authors:** Guogen Shan, Daniel Young, Le Kang

**Affiliations:** 1 Epidemiology and Biostatistics Program, Department of Environmental and Occupational Health, School of Community Health Sciences, University of Nevada Las Vegas, Las Vegas, Nevada, United States of America; 2 Division of Health Sciences, University of Nevada Las Vegas, Las Vegas, Nevada, United States of America; 3 Department of Biostatistics, Virginia Commonwealth University, Richmond, Virginia, United States of America; University of Geneva, Switzerland

## Abstract

We propose a new nonparametric test for ordered alternative problem based on the rank difference between two observations from different groups. These groups are assumed to be independent from each other. The exact mean and variance of the test statistic under the null distribution are derived, and its asymptotic distribution is proven to be normal. Furthermore, an extensive power comparison between the new test and other commonly used tests shows that the new test is generally more powerful than others under various conditions, including the same type of distribution, and mixed distributions. A real example from an anti-hypertensive drug trial is provided to illustrate the application of the tests. The new test is therefore recommended for use in practice due to easy calculation and substantial power gain.

## Introduction

The problem of statistically testing the equality of three or more populations has been studied for decades, and many efficient nonparametric tests have been proposed. Kruskal and Wallis [1] introduced a nonparametric test for a general alternative where at least two independent populations differ in median under the alternative. This test does not identify the pairwise group differences or the number of these differences. Specific ordered alternatives, such as the trend among groups, may be more interesting to practitioners and researchers. Many tests have been proposed for different types of ordering alternatives, for example, the test proposed by Mack and Wolfe [2] for an umbrella alternative, the one proposed by Fligner and Wolfe [3] for a tree alternative, the Cochran-Armitage test [4], [5] for a monotonic alternative with binary endpoints, and the Jonckheere-Terpstra (JT) test [6], [7] for a monotonic alternative with continuous endpoints.

The monotonic ordering problem with continuous endpoints occurs frequently in a wide range of statistical and medical applications [8], [9]. For example, in typical toxicity studies, the risk of adverse events that are caused, or possibly caused, by the treatment's action is often expected to rise with increasing doses. This problem has received considerable attention in the literature. After Jonckheere [6] and Terpstra [7] developed the nonparametric test for the nondecreasing ordered alternative based on the Mann Whitney (MW) testing procedure, many nonparametric tests have been developed for this problem based on the MW test or other tests. Recently, Neuhauser et al. [10] introduced a modified JT (MJT) test weighted by the distance between groups, and this test was shown to be more powerful than the JT test in small sample sizes due to the less discrete null sampling distribution. But the power gain would vanish as the sample size increases. This MJT test is a special case of the generalized JT test proposed by Tryon and Hettmansperger [8]. The Wilcoxon rank sum test was extended to the k-sample ordered problem by Cuzick [11] (referred to as the CU test) based on the the Wilcoxon rank sum test. The CU test is a special case of the linear rank test, and is a locally most powerful test for location shifts under the logistic distribution [12]. Later, Le [13] proposed a test for monotonic ordering alternatives analogous to the Kruskal Wallis test, which was shown to be equivalent to the CU test when the sample sizes were equal across groups. The numerical comparison among the JT test, the CU test, and the Le test was performed by Mahrer and Magel [14], and they found that all three tests were comparable in terms of power. Most aforementioned tests are constructed on pairwise comparisons. More recently, Terpstra and Magel [15] proposed a nonparametric test based on simultaneous comparisons with one observation from each group. In addition, interested readers are referred to Kossler [16], and Alonzo et al. [17].

In this article, we propose a new nonparametric test for the monotonic ordering problem based on the rank difference between two observations from different independent groups. The commonly used JT test statistic is calculated as the total number of pairs whose observation in the second group is greater than that in the first group. In addition to the sign of difference between two observations, the actual difference is also important to detect the ordered alternative. The actual difference can be measured by the rank difference in the nonparametric setting. The new nonparametric test captures not only the sign of the difference between observations, but also the value of the difference. We are the first to propose this new idea for detecting a monotonic ordering, and it can be readily extended to other important statistical problems.

The remainder of this article is organized as follows. In Section 2, we introduce the proposed new nonparametric rank test, derive the exact mean and variance of the test statistic under the null hypothesis, and prove the asymptotic null distribution. In Section 3, we compare the performance of the proposed test and other commonly used nonparametric tests with regard to power under a wide range of conditions. A real example from an anti-hypertensive drug trial is given to illustrate the application of the nonparametric tests in Section 4. Section 5 is given to discussion and future work.

## Nonparametric tests

The underlying distribution functions of 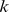 independent populations are assumed to be absolutely continuous and of the form 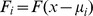, where 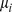 is the location parameter for the 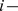
th group, 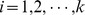. The total number of subjects in the study is 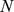, with 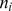 subjects in the 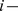
th group, and 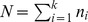. There is no difference among the 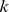 populations under the null hypothesis, and the distributions under the monotone ordering alternative differ by their location parameters 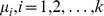. Specifically, the hypotheses are
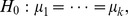
and
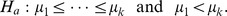



Let 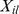 be the 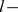
th observation in the 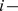
th group, and 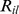 denote the rank in the combined data for the 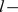
th observation in the 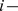
th group, where 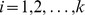 and 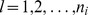. The commonly used JT test is based on the 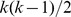 possible pairwise comparisons between two groups, and within each two group comparison the MW test statistic [18] is used. The JT test statistic is expressed as
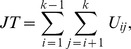
where 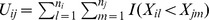 is the MW test statistic for comparing the 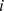-th and 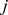-th population, 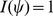 if 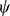 is true, and 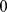 otherwise.

### 

#### 2.1 Existing nonparametric tests

In addition to the JT test, we considered three more frequently used nonparametric tests for monotonic ordering alternative problems to compare the performance with the new proposed test. They are the modified JT (MJT) test introduced by Neuhauser et al. [10], the test proposed by Terpstra and Magel [15] (referred to as the TM test), and the CU test proposed by Cuzick [11] based on the Wilcoxon rank sum test. The MJT test is a special case of generalized versions of the JT test with the weight as the distance between the group, and the test statistic is given as
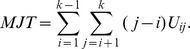



Neuhauser et al. [10] showed that the MJT test has an actual type I error closer to the nominal level and is substantially more powerful than the common JT test in small sample sizes.

Terpstra and Magel [15] introduced a nonparametric test based on the k-tuplet simultaneous comparison, not the pairwise comparison as in the JT test. A k-tuplet is constructed with one observation from each group, and the total number of k-tuplet is 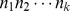. The TM test statistic is
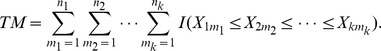



It is noted that the MW test is a special case of the TM test when 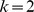.

The Wilcoxon rank sum test is one of the most popular nonparametric tests for comparing two independent populations. An extension of the Wilcoxon test was proposed by Cuzick [11]. The sum of ranks for each group is first calculated, and then the CU test statistic is computed as a weighted sum of these ranks with the weight as the group number
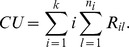



The CU test is generally more powerful than other tests under monotonic alternatives [17]. Although other tests may be considered, these four existing nonparametric tests are typically used in applications and are considered as representatives of the available tests for the monotonic ordering problem.

#### 2.2 Proposed rank test

The MW test statistic used in the JT test counts the number of pairs such that the observation from one group is greater than that from another group; however, it does not differentiate pairs using pair differences. In other words, the actual differences between observations are not well captured. We consider the actual differences to be important information that should be utilized in the testing procedure to improve the test's efficiency. Following Shan [19] for comparing two groups, the new rank based nonparametric test by incorporating the actual differences is given as
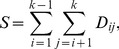
(1)where 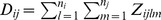, 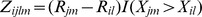 and 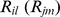 denotes the rank of the observation 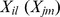 in the combined data. This new test can be considered as an extension of the sign test and the Wilcoxon rank sum test, since 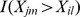 and 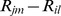 are used in the sign test and the Wilcoxon test, respectively. The exact mean and variance of the null sampling distribution are given in the following theorem.

**Theorem 2.1** *Under the null hypothesis, the new test statistic* 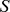 *has the mean and variance as*

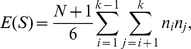

*and*

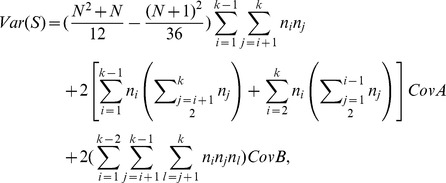
where 
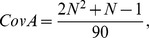
 *and* 
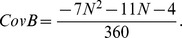


*Proof*. The calculation for the mean of 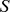 is straightforward.
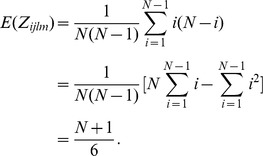



Under the null hypothesis, the expectation of 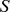 is given as
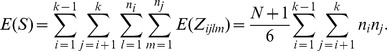



The calculation for variance is not easy and requires some effort. The variance of 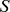 can be written as a summation of covariances,
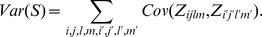



If 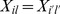, then 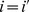 and 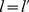; if 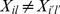, then 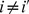 or 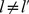. We use these notations interchangeably in this article. We consider two observations as a pair when they have the same value. Because 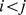 and 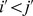, one observation from a pair is from 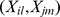 and the other is from 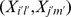.

The covariance is non-zero only when at least one pair exists in the observations 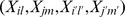. The maximum number of pairs in 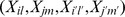 is two, with 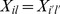 and 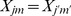. Then 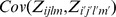 is the variance of 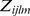.
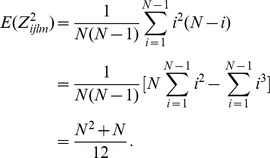



Thus, the 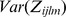 under the null hypothesis is expressed as
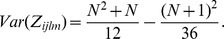



When only one pair exists in 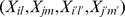, there are four possible outcomes: (a) 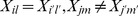, (b) 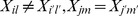, (c) 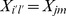 and (d) 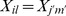. In cases (a) and (b), the observations 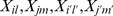 are either from two groups where the unpaired two observations are from one group and the pair is from the other, or from three groups where the pair is from either the first group or the third group after the groups have been sorted.
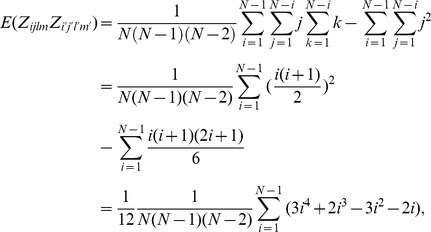



The first type of covariance in the case with only one pair is
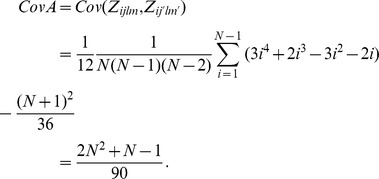



In cases (c) and (d), the observations 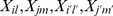 are from three different groups and the pair is from the second group (the middle group) after sorting the groups.
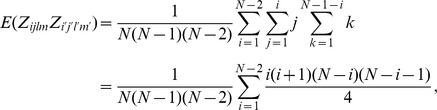



Then, the second type of covariance in the case with only one pair is given as
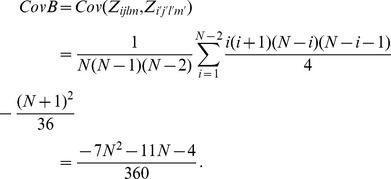



In the case with no pair in the observations 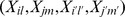, 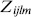 and 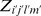 are independent, and 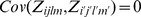.

Therefore, the variance of 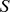 is given as
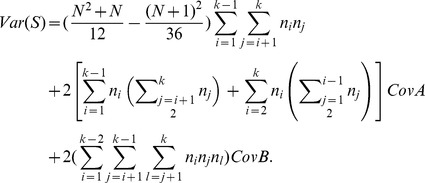
□

The standardized test statistic of 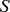 is
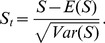
(2)


The following theorem shows the asymptotic normality of the test statistic 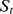 under the null hypothesis.

**Theorem 2.2** *When* 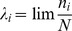 *exists,* 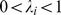 *, the proposed test* 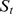 *has an asymptotic standard normal distribution as* 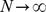 *and* 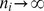 *.*

*Proof*. Let 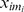 be the 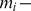
th observation in the 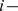
th group, where 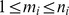. Define
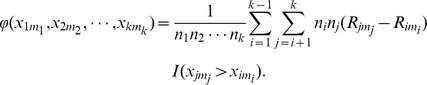



It should be noted that
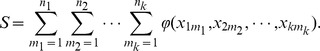



By applying the results of the Problem 42 in the Appendix of Lehmann [20], 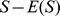 asymptotically follows a normal distribution without scaling by the standard deviation, which can be proven by projecting the test statistic 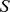 onto a sum of independent random variables [21] and then applying the central limit theorem.□

The new proposed test can be performed by comparing 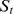 with appropriate quantile of standard normal distribution. For example, at the significance level of 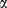, the null hypothesis will be rejected in the favor of an increasing ordered alternative if 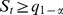, where 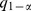 is the upper 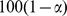 percentile of the standard normal distribution.

**Figure 1:**
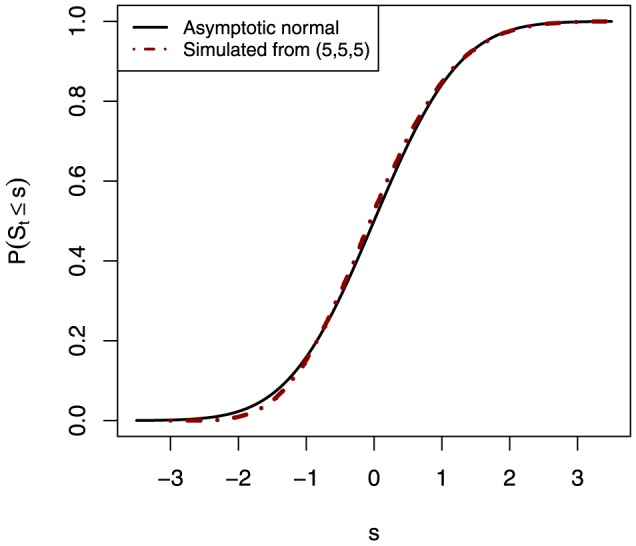
The cumulative distribution function based on the asymptotic distribution, and based on the Monte Carlo simulation based exact distribution for 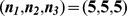.

The asymptotic cumulative distribution function (CDF) and the Monte Carlo simulation based exact distribution of 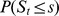 for 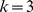, 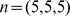 are displayed in Figure 1. The simulated exact distribution was based on 20,000 iterations from the standard normal distribution for each group. As seen in the figure, the exact permutation distribution approximates the asymptotic distribution well.

## Numerical study

We conduct extensive exact Monte Carlo simulation studies to compare the five tests: 1): the JT test; 2) the MJT test; 3) the TM test; 4) the CU test; and 5) the new proposed test. The nominal level is set to be 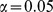. In order to make a fair comparison between tests and avoid unsatisfied type I error rate control for tests using asymptotic distributions, exact permutation approach is used with data simulated from standard normal distributions with the same location and scale, e.g., 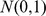. Total 20,000 iterations are utilized to obtain the 95% cutpoint, and these 20,000 simulated data is used for all the methods. For given the number of group and sample size within each group, the 95% cutpoint for each test is computed from the same simulated null distribution. In other words, the simulated null distribution under each configuration, is used multiple times to cacluate the cutpoint for each test. The same rule is applied to the simulated alternative distribution for power comparison. This procedure would reduce the bias of cutpoint and power estimates between tests, and makes a fair comparison between them.

The number of groups with 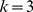 and 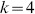 are considered in the power comparison. The simulated power is calculated as the proportion of iterations whose test statistic falls in the rejection region based on 10,000 simulations. Sample sizes 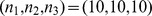, 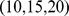, 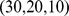, and 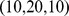 are examined, and five alternatives are considered for normal distributions: four with a unit variance 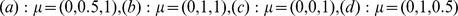, and one with different variances 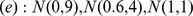. The parameters for alternative distributions (a), (b), (c), and (d) are also used for the t distribution with df = 3 of the form 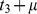. In addition to symmetric distributions, we also consider a skewed distribution, exponential distribution, and a mixed distribution of normal distribution and exponential distribution. We consider similar distributions for the case of 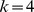, but with the sample sizes 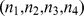: (8,8,8,8), (10,6,6,10), (20,20,10,10), and (10,20,10,20), and three alternatives: 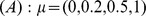, 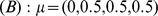, and 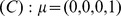. The power comparison between the five tests is examined for each configuration of sample size and alternative hypothesis.

The simulated power under normal distributions for 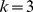 is shown in Table 1. The actual sizes were obtained by simulating samples from standard normal distributions using the simulated 95% cutpoint. Simulated sizes are generally closer to the nominal level across the tests and sample sizes considered. We observe that the MJT test and the test due to Cuzick have the same power, which is also observed under other distributions. Although we do not theoretically prove that both tests have the same power using exact permutation test, it may be the case that they are equivalent to each other. For this reason, we only present one of them in the following power comparison results. The TM test has some power gain compared to other tests under the convex shape alternative (c) with decreasing sample sizes across groups. We have seen this trend from the other three distributions. The TM test has some power advantage as compared to others under the normal distribution with unequal variances. In all other configurations, the power of the TM test is lower than that of other tests. Out of the total 20 configurations from the alternative (a)-(e) and four difference sample sizes, the new test has more power than the JT test in 19 cases, and is at least as powerful as the CU test in 15 cases.

**Table 1:** Simulated size and power study based on normal distribution for 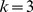.

		Tests				
Distribution	Sample sizes	JT	MJT	TM	CU	New
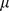 = (0,0,0)	(10,10,10)	0.047	0.047	0.049	0.047	0.051
	(10,15,20)	0.044	0.045	0.048	0.045	0.048
	(30,20,10)	0.045	0.048	0.048	0.048	0.047
	(10,20,10)	0.051	0.053	0.051	0.053	0.055
(a) 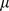 = (0,0.5,1)	(10,10,10)	0.662	0.663	0.601	0.663	**0.679**
	(10,15,20)	0.792	**0.804**	0.723	**0.804**	0.804
	(30,20,10)	0.852	0.860	0.786	**0.860**	0.856
	(10,20,10)	0.681	0.688	0.652	0.688	**0.695**
(b) 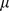 = (0,1,1)	(10,10,10)	0.637	0.649	0.527	0.649	**0.684**
	(10,15,20)	0.607	**0.682**	0.649	**0.682**	0.653
	(30,20,10)	0.968	0.951	0.695	0.951	**0.971**
	(10,20,10)	0.650	0.665	0.569	0.665	**0.701**
(c) 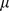 = (0,0,1)	(10,10,10)	0.634	0.645	0.519	0.645	**0.683**
	(10,15,20)	0.886	0.866	0.625	0.866	**0.902**
	(30,20,10)	0.578	0.687	**0.724**	0.687	0.614
	(10,20,10)	0.660	0.670	0.571	0.670	**0.710**
(d) 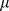 = (0,1,0.5)	(10,10,10)	0.221	0.234	0.144	0.234	**0.290**
	(10,15,20)	0.100	**0.161**	0.152	**0.161**	0.143
	(30,20,10)	0.790	0.688	0.209	0.688	**0.830**
	(10,20,10)	0.229	0.245	0.151	0.245	**0.310**
(e) N(0,9), N(0.6,4), N(1,1)	(10,10,10)	0.131	0.122	**0.186**	0.116	0.153
	(10,15,20)	0.162	0.164	0.229	0.166	**0.244**
	(30,20,10)	0.116	0.112	**0.194**	0.105	0.096
	(10,20,10)	0.134	0.128	**0.180**	0.125	0.142

The power study under other distributions for 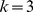 are shown in Table 2 for the t alternative and in Table 3 for the exponential distribution. The exponential distribution is examined as an example of skewed distributions, with mean values: 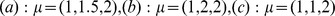, and (d): 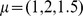. The new test has the highest power in 13 of the 16 configurations under the t distribution, and 12 under the exponential distribution. The new test is generally more powerful than other tests under the linear alternative (a) for the t distribution.

**Table 2:** Simulated power study based on t distributions with df = 3 of the form 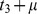 for 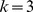.

		Tests			
Distribution	Sample sizes	JT	TM	CU	New
(H0) 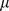 = (0,0,0)	(10,10,10)	0.045	0.051	0.045	0.051
	(10,15,20)	0.047	0.048	0.049	0.051
	(30,20,10)	0.046	0.048	0.045	0.050
	(10,20,10)	0.050	0.052	0.049	0.053
(a) 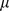 = (0,0.5,1)	(10,10,10)	0.503	0.461	0.504	**0.525**
	(10,15,20)	0.630	0.584	0.641	**0.643**
	(30,20,10)	0.716	0.655	0.725	**0.726**
	(10,20,10)	0.510	0.491	0.506	**0.523**
(b) 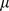 = (0,1,1)	(10,10,10)	0.485	0.416	0.491	**0.527**
	(10,15,20)	0.472	0.517	**0.537**	0.511
	(30,20,10)	0.872	0.579	0.841	**0.888**
	(10,20,10)	0.498	0.443	0.502	**0.537**
(c) 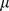 = (0,0,1)	(10,10,10)	0.475	0.410	0.484	**0.520**
	(10,15,20)	0.736	0.515	0.713	**0.762**
	(30,20,10)	0.436	**0.584**	0.529	0.468
	(10,20,10)	0.499	0.444	0.503	**0.538**
(d) 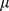 = (0,1,0.5)	(10,10,10)	0.177	0.132	0.186	**0.225**
	(10,15,20)	0.098	0.140	**0.146**	0.128
	(30,20,10)	0.623	0.183	0.524	**0.676**
	(10,20,10)	0.181	0.136	0.188	**0.234**

**Table 3:** Simulated power study based on exponential distribution for 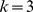.

		Tests			
Distribution	Sample sizes	JT	TM	CU	New
(H0) 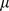 = (1,1,1)	(10,10,10)	0.046	0.052	0.047	0.051
	(10,15,20)	0.047	0.052	0.048	0.051
	(30,20,10)	0.048	0.053	0.049	0.050
	(10,20,10)	0.050	0.052	0.050	0.055
(a) 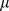 = (1,1.5,2)	(10,10,10)	0.351	0.321	0.352	**0.367**
	(10,15,20)	0.425	0.405	**0.438**	0.426
	(30,20,10)	0.543	0.446	0.542	**0.565**
	(10,20,10)	0.356	0.331	0.353	**0.366**
(b) 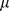 = (1,2,2)	(10,10,10)	0.324	0.262	0.328	**0.351**
	(10,15,20)	0.320	0.321	**0.363**	0.335
	(30,20,10)	0.686	0.370	0.642	**0.720**
	(10,20,10)	0.343	0.277	0.344	**0.361**
(c) 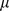 = (1,1,2)	(10,10,10)	0.336	0.323	0.342	**0.362**
	(10,15,20)	0.538	0.409	0.512	**0.552**
	(30,20,10)	0.311	**0.454**	0.372	0.338
	(10,20,10)	0.349	0.342	0.348	**0.382**
(d) 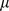 = (1,2,1.5)	(10,10,10)	0.152	0.104	0.160	**0.180**
	(10,15,20)	0.106	0.121	**0.138**	0.121
	(30,20,10)	0.483	0.148	0.416	**0.534**
	(10,20,10)	0.149	0.103	0.155	**0.171**

We also compare the tests with mixed distributions for 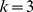 in Table 4. The mixed distribution considered here is: normal distribution for the first group, and exponential distributions for the second group and the third group, with mean values: 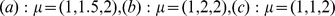, and (d): 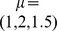. In the normal distribution, univariate variance is used. When the same distributions are used for each group as aforementioned, the actual type I error rates are close to the nominal level. However, in the mixed distribution, the actual type I error rates are conservative for the case considered, especially in the case with decreasing sample sizes. Nevertheless, the new test has more power than other tests in 12 out of the total 16 configures.

**Table 4:** Simulated power study based on the mixed distribution for 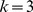.

		Tests			
Distribution	Sample sizes	JT	TM	CU	New
(H0) 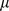 = (1,1,1)	(10,10,10)	0.029	0.045	0.028	0.030
	(10,15,20)	0.029	0.048	0.028	0.030
	(30,20,10)	0.017	0.032	0.018	0.015
	(10,20,10)	0.032	0.043	0.030	0.034
(a) 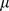 = (1,1.5,2)	(10,10,10)	0.251	0.240	0.252	**0.265**
	(10,15,20)	0.329	0.300	**0.333**	0.328
	(30,20,10)	0.370	0.335	0.380	**0.381**
	(10,20,10)	0.266	0.255	0.265	**0.273**
(b) 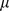 = (1,2,2)	(10,10,10)	0.243	0.200	0.247	**0.266**
	(10,15,20)	0.240	0.232	**0.267**	0.243
	(30,20,10)	0.524	0.278	0.491	**0.549**
	(10,20,10)	0.248	0.205	0.250	**0.257**
(c) 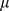 = (1,1,2)	(10,10,10)	0.253	0.267	0.256	**0.281**
	(10,15,20)	0.444	0.327	0.414	**0.463**
	(30,20,10)	0.186	**0.345**	0.242	0.198
	(10,20,10)	0.253	0.271	0.251	**0.294**
(d) 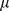 = (1,2,1.5)	(10,10,10)	0.097	0.074	0.103	**0.118**
	(10,15,20)	0.069	0.078	**0.086**	0.076
	(30,20,10)	0.322	0.092	0.271	**0.351**
	(10,20,10)	0.099	0.070	0.103	**0.111**

The power comparison results for 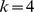 are shown in Tables 5, 6, 7, and 8 for the normal distribution, the t distribution, the exponential distribution, and the mixed distribution. The mixed distribution is the one with normal distributions 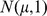 for the first two groups, and exponential distributions 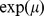 with mean 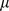 for the last two groups. As can be seen from these tables, the new test generally has more power than all other existing tests, and is almost uniformly more powerful than the commonly used JT test.

**Table 5:** Simulated size and power study based on normal distribution for 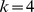.

		Tests			
Distribution	Sample sizes	JT	TM	CU	New
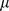 = (0,0,0,0)	(8,8,8,8)	0.050	0.053	0.053	0.054
	(10,6,6,10)	0.048	0.049	0.047	0.048
	(20,20,10,10)	0.052	0.050	0.050	0.053
	(10,20,10,20)	0.049	0.048	0.050	0.051
(A) 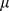 = (0,0.2,0.5,1)	(8,8,8,8)	0.623	0.497	0.633	**0.638**
	(10,6,6,10)	0.680	0.494	0.685	**0.693**
	(20,20,10,10)	0.778	0.686	**0.804**	0.784
	(10,20,10,20)	0.891	0.684	0.893	**0.898**
(B) 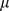 = (0,0.5,0.5,0.5)	(8,8,8,8)	0.205	0.168	0.212	**0.220**
	(10,6,6,10)	0.253	0.173	0.254	**0.268**
	(20,20,10,10)	0.435	0.241	0.382	**0.452**
	(10,20,10,20)	0.233	0.237	0.239	**0.243**
(C) 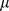 = (0,0,0,1)	(8,8,8,8)	0.522	0.370	0.540	**0.564**
	(10,6,6,10)	0.634	0.374	0.638	**0.672**
	(20,20,10,10)	0.540	0.522	**0.656**	0.575
	(10,20,10,20)	0.894	0.516	0.903	**0.916**

**Table 6:** Simulated size and power study based on t distribution with with df = 3 of the form 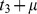 for 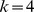.

		Tests			
Distribution	Sample sizes	JT	TM	CU	New
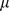 = (0,0,0,0)	(8,8,8,8)	0.048	0.050	0.050	0.051
	(10,6,6,10)	0.047	0.050	0.050	0.052
	(20,20,10,10)	0.053	0.052	0.053	0.054
	(10,20,10,20)	0.050	0.054	0.052	0.052
(A) 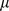 = (0,0.2,0.5,1)	(8,8,8,8)	0.464	0.365	0.473	**0.481**
	(10,6,6,10)	0.521	0.375	0.536	**0.542**
	(20,20,10,10)	0.609	0.549	**0.643**	0.620
	(10,20,10,20)	0.742	0.552	0.745	**0.751**
(B) 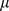 = (0,0.5,0.5,0.5)	(8,8,8,8)	0.169	0.143	0.174	**0.180**
	(10,6,6,10)	0.199	0.142	0.211	**0.220**
	(20,20,10,10)	0.323	0.191	0.290	**0.340**
	(10,20,10,20)	0.193	0.194	0.195	**0.198**
(C) 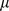 = (0,0,0,1)	(8,8,8,8)	0.390	0.287	0.407	**0.426**
	(10,6,6,10)	0.470	0.288	0.484	**0.510**
	(20,20,10,10)	0.404	0.401	**0.497**	0.431
	(10,20,10,20)	0.759	0.425	0.764	**0.783**

**Table 7:** Simulated size and power study based on exponential distribution for 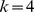.

		Tests			
Distribution	Sample sizes	JT	TM	CU	New
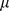 = (1,1,1,1)	(8,8,8,8)	0.050	0.052	0.051	0.053
	(10,6,6,10)	0.048	0.052	0.052	0.054
	(20,20,10,10)	0.050	0.050	0.047	0.050
	(10,20,10,20)	0.046	0.046	0.048	0.048
(A) 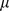 = (1,1.2,1.5,2)	(8,8,8,8)	0.333	0.277	0.344	**0.349**
	(10,6,6,10)	0.351	0.262	0.366	**0.371**
	(20,20,10,10)	0.445	0.383	**0.464**	0.457
	(10,20,10,20)	0.513	0.384	**0.523**	0.521
(B) 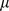 = (1,1.5,1.5,1.5)	(8,8,8,8)	0.145	0.115	0.154	**0.160**
	(10,6,6,10)	0.162	0.112	0.174	**0.184**
	(20,20,10,10)	0.265	0.143	0.239	**0.278**
	(10,20,10,20)	0.152	0.140	**0.158**	0.157
(C) 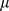 = (1,1,1,2)	(8,8,8,8)	0.285	0.251	0.293	**0.307**
	(10,6,6,10)	0.322	0.241	0.336	**0.346**
	(20,20,10,10)	0.293	**0.358**	0.348	0.310
	(10,20,10,20)	0.552	0.362	0.564	**0.570**

**Table 8:** Simulated size and power study based on the mixed distribution for 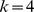.

		Tests			
Distribution	Sample sizes	JT	TM	CU	New
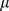 = (1,1,1,1)	(8,8,8,8)	0.027	0.023	0.028	0.029
	(10,6,6,10)	0.022	0.021	0.024	0.026
	(20,20,10,10)	0.018	0.016	0.019	0.019
	(10,20,10,20)	0.016	0.016	0.015	0.016
(A) 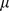 = (1,1.2,1.5,2)	(8,8,8,8)	0.230	0.182	0.234	**0.240**
	(10,6,6,10)	0.252	0.180	0.262	**0.267**
	(20,20,10,10)	0.336	0.262	0.343	**0.347**
	(10,20,10,20)	0.340	0.245	**0.344**	**0.344**
(B) 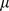 = (1,1.5,1.5,1.5)	(8,8,8,8)	0.085	0.063	0.086	**0.091**
	(10,6,6,10)	0.098	0.060	0.101	**0.106**
	(20,20,10,10)	0.189	0.078	0.147	**0.206**
	(10,20,10,20)	0.057	0.059	0.056	**0.060**
(C) 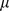 = (1,1,1,2)	(8,8,8,8)	0.194	0.143	0.201	**0.213**
	(10,6,6,10)	0.216	0.147	0.226	**0.241**
	(20,20,10,10)	0.190	0.196	**0.236**	0.201
	(10,20,10,20)	0.385	0.194	0.391	**0.395**

## Example

A clinical trial for an antihypertensive drug [22] is provided to illustrate the use of the discussed tests. The primary objective of the study was to examine the effect of the selected doses on diastolic blood pressure by measuring the mean reduction in diastolic blood pressure. Patients with hypertension were randomized into four groups with different dose levels, 0, 10, 20, and 40 mg/day, where the group with 0 mg/day was the placebo group. The number of patients in each group were 17, 17, 18, and 16, respectively. The complete data can be found at the companion web site of the book by Dmitrienko et al. [22]. The mean reduction in diastolic blood pressure was expected to increase as the daily dose of the antihypertensive drug increased. Therefore, a monotonic increasing alternative is appropriate for this problem: 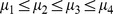. The permutation p-values for the JT test, the MJT test, the TM test, the CU test, and the new test are 0.00210, 0.00270, 0.01245, 0.00270, and 0.00250, respectively. At the significance level of 0.05, these five tests share the same conclusion that the relationship between the dose usage and the mean reduction in diastolic blood pressure is positive. The program is written in R, and is available from the author's website: https://faculty.unlv.edu/gshan/. You may contact the corresponding author for any questions you may have.

## Conclusion

In this article we propose a new powerful nonparametric test, based on the rank difference between observations, for the monotonic ordering alternative problem in k-sample problem. The rank difference between observations for two groups is analogous to the two sample t test when the parametric assumptions are satisfied. The positive rank differences used in the test statistic are motivated by the idea of the sign test. We derive the asymptotic distribution of the new test statistic and studied the convergence rate of the simulation based exact distribution to the asymptotic distribution. The power comparison between the new test and other existing tests shows that the new test is generally more powerful than other tests for various distributions. We would recommend using the new test in practice due to substantial power gain.

The asymptotic distribution of the new test statistic was derived with continuous endpoints. No ties occur in continuous data. For ordinal and binary data, one has to consider the frequency of ties in the data, and the variance of the new test needs to be investigated. However, for given data, permutation based or simulation based approaches are readily employed for the p-value calculation. The application of the new test for ordinal or binary data is considered for future work. Other alternative hypotheses may be studied, such as the general alternative [1], the umbrella alternative [2], and the tree alternative [3]. An extension of the new test in exact testing framework [23], [24], [25], [26] and for repeated data from randomized block designs are also interesting.
